# Simple method for identification of women at risk of gestational diabetes mellitus in Arusha urban, Tanzania

**DOI:** 10.1186/s12884-022-04838-1

**Published:** 2022-07-06

**Authors:** Safiness Simon Msollo, Haikael David Martin, Akwilina Wendelin Mwanri, Pammla Petrucka

**Affiliations:** 1 Depertment of Food Technology, Nutrition and Consumer Sciences, Sokoine University of Agriculture, Morogoro, Tanzania; 2 School of Life Sciences, Nelson Mandela African Institution of Science and Technology, Arusha, Tanzania; 3 College of Nursing, University of Saskatchewan, Saskatoon, Canada

**Keywords:** Risk score, Checklist, Gestational diabetes mellitus, Tanzania

## Abstract

**Background:**

Screening for gestational diabetes mellitus in Tanzania is challenged by limited resources. Therefore, this study aimed to develop a simple method for identification of women at risk of gestational diabetes mellitus in Arusha urban, Tanzania.

**Methods:**

This study used data from a cross sectional study, that was conducted between March and December 2018 in Arusha District involving 468 pregnant women who were not known to have diabetes before pregnancy. Urine glucose was tested using urine multistics and blood glucose levels by Gluco-Plus™ and diagnosed in accordance with the World Health Organization’s criteria. Anthropometrics were measured using standard procedures and maternal characteristics were collected through face-to-face interviews using a questionnaire with structured questions. Univariate analysis assessed individual variables association with gestational diabetes mellitus where variables with *p*-value of < 0.05 were included in multivariable analysis and predictors with *p*-value < 0.1 remained in the final model. Each variable was scored based on its estimated coefficients and risk scores were calculated by multiplying the corresponding coefficients by ten to get integers. The model’s performance was assessed using c-statistic. Data were analyzed using Statistical Package for Social Science™.

**Results:**

The risk score included body fat ≥ 38%, delivery to macrosomic babies, mid-upper arm circumference ≥ 28 cm, and family history of type 2 diabetes mellitus. The score correctly identified 98% of women with gestational diabetes with an area under the receiver operating characteristic curve of 0.97 (95% CI 0.96–0.99, *p* < 0.001), sensitivity of 0.98, and specificity of 0.46.

**Conclusion:**

The developed screening tool is highly sensitive and correctly differentiates women with and without gestational diabetes mellitus in a Tanzanian sub-population.

## Introduction

Diabetes is the most common metabolic disorder affecting pregnancy and its prevalence increases with overweight and obesity [1]. Gestational diabetes mellitus (GDM) results from pregnancy-induced changes in maternal glucose metabolism and insulin sensitivity whereby demand for insulin production on the mother’s pancreas increases as pregnancy grows [2, 3]. During normal pregnancy, mother’s body undergoes a series of physiological changes to support the demand of the growing fetus including; adaptation of the cardiovascular, renal, hematologic, respiratory, and metabolic systems [3]. The important metabolic adaptation is insulin sensitivity, which changes with the increasing needs over the course of pregnancy. During the early stage of gestation, insulin sensitivity increases, which promote glucose uptake into the adipose stores to prepare for the future energy demands of the pregnancy however, as pregnancy proceeds, placental hormones promote a state of insulin resistant (IR) [4, 5].

Insulin resistance in pregnancy is the failure of a precise concentration of insulin to affect an anticipated biological response of nutrient metabolism caused by increased maternal adiposity and insulin-desensitizing influences of placental hormones [6]. This condition arises when pancreatic β-cells are not able to release adequate insulin to counterbalance IR which starts at the middle of pregnancy and continues up to the third trimester [2]. As pregnancy continues to grow, the release of placental hormones, including estrogen, progesterone, cortisol, and lactogen increases as well, which hinder insulin to function normally and slowly reducing insulin sensitivity to half (fifty percent) of the anticipated value [7]. These placental hormones can enlarge the islets of Langerhan cells and/or the hyperplasia of the pancreatic β-cells to increase the release of extra insulin, leading to compensated hyperinsulinaemia [8, 9].

In addition to these placental hormones, several metabolic variations during pregnancy increase adipose tissues that may produce many adipocytokines which can act like hormones involved in regulation of maternal metabolism and gestational IR like adipokines (tumor necrosis factor [TNF]-alpha and leptin). These can cause impairments in insulin signaling leading to IR state [10–12]. The inadequate insulin secretion to balance the reduced insulin sensitivity may result into GDM [12].

In most instances, women meet the increased insulin demand, but failure to accommodate potentiates poor glycemic control [2, 12]. Gestational diabetes mellitus normally disappears after delivery however, if not diagnosed and managed, can cause short-and long-term effects to the mother and her newborn such as pregnancy induced hypertension (PIH) and type 2 diabetes mellitus (T2DM) within 12 weeks after delivery [13]. Women with GDM experience; miscarriage, preterm birth, stillbirth and/or neonatal death [14, 15]. Furthermore, these women can deliver a macrosomic baby (> 4 kg at birth) and increase birth trauma [16]. Macrosomic infants are at risk of hypoglycemia soon after birth because their bodies continue producing extra insulin in response to the mothers’ excess glucose [17]. However, if a woman is identified and/or confirmed to have GDM or the associated risk factors, medical nutrition therapy, self-monitoring of blood glucose, physical activity and food plan can be done for good glycemic control, effective lactation, and infant health. Therefore, during pregnancy women with GDM should be educated that glucose intolerance may not be temporary, can be modified by behavior changes hence, postpartum testing is very important [18].

The prevalence of different forms of GDM in Tanzania varies depending on locality whereby 16.2% was reported in Arusha, 18% in Dar es Salaam, and about 20% in Kilimanjaro Region using the WHO [2] diagnostic criteria [19–21]. The risk of developing GDM increases with family history of diabetes, maternal obesity/overweight as well as macrosomic delivery, stillbirth, preterm delivery and neonatal death in previous pregnancies [22]. In addition, low level of physical activities, poor dietary intake and extreme pregnancy weight gain expose women to higher risk of GDM [23, 24].

Knowledge on these risk factors, provide useful information for health care providers in educating women on appropriate GDM screening and management [25]. Timely identification of women at risk of GDM for further testing of the few selected women minimizes inconvenience, time, and healthcare costs [2]. Cost-effectiveness methods need to be developed and individualized by country for optimal testing and managing GDM given their specific burden of disease and resource constraints [3]. In Tanzania, due to limited resources, most women are not screened for hyperglycemia before, during, and/or after pregnancy and, even if screened, they are subject to glucose testing in urine despite its poor sensitivity [26].

It should be known that countries select their own methods and criteria for GDM screening due to resource constraints and situational applicability. Some guidelines recommend universal screening by an oral glucose tolerance test (OGTT) and/or fasting blood glucose test [2]; and others exclude the low-risk women from testing [27]. There is lack of evidence on how universal strategies improve maternal/child health compared to selective strategies, given the increase in associated costs, clinician workloads, potential inconveniences, and competing priorities [28].

Several GDM selective screening methods have been developed in different settings and population groups. However, most strategies have been developed and tested in Caucasian and Asian populations [29–32] with few based in African populations [33–36]. Generalization of the developed risk scores is impossible due to differences in research design, selection of participants, gestational age at screening, and diagnosis criteria used. The evidence from eight published prediction models, tested in a South African population shown that, the models performed poorly as screening tools for GDM as compared to their origin populations [34]. This may be attributed to determinants of GDM which vary across settings due to differences in body composition, lifestyle, and genetic predisposition [37].

Furthermore, the previous risk score developed in Tanzania, involved mid-upper arm circumference (MUAC) ≥ 28 cm, previous stillbirth, and family history of type 2 diabetes mellitus (T2DM) as significant risk factors for GDM. This calls for further development of the tool that involves more risk factors such as maternal age, history of macrocosmic babies, pre-pregnancy body mass index (BMI), hypertension, and pregnancy weight gain [35].

Body mass index (BMI) is a frequently used indicator for assessing nutrition status of pregnant women but cannot clearly differentiate fat mass from lean body mass [38] and most of the women start antenatal clinic (ANC) late without knowing their body weights, which complicates estimations of their BMI [39]. Also, the fetal mass and amniotic fluid contain an unspecified part of the overall body mass of the mother. Therefore, there is a need to consider the less explored body fat percentage (BF%) that is measured using bioelectrical impedance analysis (BIA) as it is known to be a safe, accurate, and reliable method for assessing nutrition status [40]. Furthermore, mid upper arm circumference (MUAC) which is cheap and simple to measure, can be used instead of BMI due to its constancy throughout pregnancy and highly associated with pre-pregnancy BMI [41, 42].

Therefore, the current study aimed to construct a more suitable selective screening strategy for Tanzania’s antenatal care (ANC) settings that focuses on opportunities to enhance self-care to ensure better neonatal and maternal outcomes.

## Methods

The study was done as part of a cross-sectional study that was conducted in urban areas of Arusha City between March and December 2018 among pregnant women attending ANC at Ngarenaro and Kaloleni Health Centers. The study involved women in their second and third trimesters without diabetes before pregnancy while women who were known and confirmed to have diabetes and under managements were excluded from the study [19]. The eligible women were randomly selected until a total of 468 pregnant women were selected to participate. This sample size was obtained using the formula for prevalence studies [43] where prevalence (i.e., p) was assumed to be 50% and a non-response rate of 20% was selected due to limited national data for prevalence of GDM [44]. The study was approved by the Tanzania National Institute for Medical Research (NIMR) with a reference number NIMR/HQ/R.8a/VoLIX/2694. Participants signed an informed consent which explained the aim, procedures, benefits, and potential effects of the study.

### Assessment of demographic characteristics and selected risk factors for GDM

Recalled information with respect to pre-pregnancy weight, previous birth modalities (i.e., caesarean section or normal delivery), family history of T2DM, previous history of GDM, and previous delivery of neonates with ≥ 4 kg at birth were collected through face-to-face interviews using a questionnaire with structured questions. Other clinical and maternal characteristics, such as age, histories of stillbirth, and neonatal death, gravidity, education level, occupation, marital status, and weight during the first antenatal visit were obtained from the participants’ ANC records. Post-delivery information from the index pregnancy were collected by the skilled birth attendants using a short questionnaire which was attached to every participant’s ANC card when the study began. Birth outcomes in the index pregnancy included gestational weeks at delivery, birth modality, stillbirth, and/or neonatal death, infant’s weight at birth, and any abnormalities.

### Blood samples collection for testing GDM

Participants were requested to fast overnight (8–12 h) before blood samples collection. The next day, fasting capillary blood samples were taken and tested for GDM followed by consumption of 75 g of glucose powder dissolved in 300 ml of water. Fasting blood glucose and 2 h OGTT were tested using Gluco-plus™ (Glucoplus Inc. 2323 Halpern, Ville St. Laurent, Quebec, Canada). The capillary plasma glucose values obtained were converted to venous plasma glucose using the regression equation that is developed for diabetes screening in low resource areas [45]. Urine samples were also collected using disposable hospital urine sample containers (60 ml) in the morning and tested for glucose within one hour using multi-sticks with color sensitive pads (Urine strips 388–25, Gomo-ro, Gimhae-si, Gyeongsangnam-do, 621–881, Korea).

### Anthropometric assessments

Mid-upper arm circumference was measured using a non-stretchable standard tape. Weight was measured with minimal clothing and without shoes using a digital bathroom weighing scale (SECA-Germany), where two measurements were taken and recorded to the nearest 0.1 kg. The infant’s weight at birth was measured by the normal infant weighing scale used in the ANC [46]. Height was measured in duplicate using a stadiometer (Shorr Productions, Maryland USA). This height and the recalled pre-pregnancy weight were used to calculate pre-pregnancy BMI. Body fat percentage was determined using a bioelectric impedance analyzer (Tanita TBF 105 Fat Analyzer™) which included adjustments for age, weight and height. Body fat percentage and MUAC are simple to measure and can complement the missing information with regards to recalled weight where most of the pregnant women cannot remember their weight before pregnant and are late to start ANC which makes the use of early pregnancy weight impossible.

Blood pressure was measured using a GT-868UF Geratherm™ machine after a participant had relaxed for ten minutes before the actual measurement where two measurements were done at an interval of five minutes and the average was recorded. Blood pressure was classified using the Standard treatment guidelines and essential medicines list categories of systolic 140 to 159 mmHg or diastolic 90 to 99 mmHg [47].

### Development of GDM risk scores

Through binary logistic regression, univariate analysis of each variable in relation to GDM was done to assess their individual contributions in prediction of GDM. All variables with a *p*-value of < 0.05 were entered in a model. Multivariable analysis was performed with stepwise backwards elimination [48] and all non-significant predictors (*p* value > 0.1) were eliminated at this stage and a new model with significant predictors was set. For the model to be applicable, each risk factor was scored based on the estimated coefficients of regression whereby the increase in number of the scores indicates high risk of GDM [30]. The risk scores were calculated, for each risk factor based on their corresponding coefficients multiplied by ten [19] to remove decimals to get integers for easier interpretation and application by health care providers and women themselves.

Performance of the model was assessed by discrimination assessment using the c-statistic for binary outcomes. Three other models were developed based on OGTT, fasting blood glucose and urine tests results to compare their performance with that of the risk score model. The comparisons were done using the c-statistic for binary outcomes where area under the receiver operating characteristic curve (AUC) for each model was determined as well as sentstivity and specificity.

### Data analysis

Data were analyzed using the Statistical Package for Social Science™ (SPSS™) Version 20 through which descriptive statistics, such as frequency, mean, and percentage, were obtained. Blood glucose values were dichotomized into either having GDM or not having GDM, and univariate analysis was done for the variables associated with GDM to obtain crude odd ratios [49]. Multiple logistic regression analysis explored whether different factors had significant association with GDM. Statistical inference was based on 95% confidence intervals (CIs) and significance at *p* value ≤ 0.05 for univariate analysis and *p* < 0.1 for the final risk score model to avoid loss of important variables which contribute to the development of GDM. Lastly, the model was simplified into a risk factors checklist for operationalization to enhance self-identifications.

## Results

A total of 468 pregnant women participated in the study. The mean age of the studied women was 28 ± 5.84 years, of which 65.6% were ≥ 25 years old. Most started ANC at the mean gestational age of 18 ± 5.62 weeks. At the commencement of this study, the mean gestational age was 28.5 ± 3.82 weeks. About 62% (*n* = 354) of the women were ≥ 28 weeks of gestation at the time of entry to the study with 38% (*n* = 177) being second or third gravidity (Table 1).

**Table 1:** Demographic characteristics of participants during pregnancy

Respondent Variables	Frequency	Percent	Mean (± SD)
Gestational age at first ANC visit			
< 12 weeks	57	12.2	
12–24 weeks	363	77.6	18 (SD ± 5.62)
25–36 weeks	48	10.2	
Gestational age at study commencement			
24–28 weeks	291	62.2	28 (SD ± 3.82)
> 28 weeks	177	37.8	
Gravidity			
Prime	142	30.3	
Second and third	236	50.4	3 (SD ± 1.20)
Fourth and above	90	19.2	
Age			
< 25 years	164	35.0	28 (SD ± 5.84)
≥ 25 years	304	65.0	

The mean of the self-reported pre-pregnancy weight for the women who remembered their weight before pregnancy was 67 ± 12.5 kg with a BMI of 25.7 ± 5.7. The measured mean height was 159 ± 6.3 cm, weight during pregnancy was 69 ± 12.9, body fat 33.7 ± 7.2%, and MUAC 27 ± 3.8 cm. Pre-pregnancy BMI was determined with 25.2% (*n* = 60) classified as overweight and 22.7% (*n* = 54) as obese. About 36% (*n* = 164) of the women had MUAC ≥ 28 cm which is indicative of overweight or obesity. Twenty percent (*n* = 94) of the women were hypertensive and 20.3% (*n* = 95) had edema (Table 2).

**Table 2:** Anthropometric measurements

Respondent Variables	Frequency	Percent	Mean (± SD)
Body fat percentage	468	100.0	33.4 (SD ± 7.8)
Self-reported pre-pregnancy weight (kg)	240	51.3	67 (SD ± 12.5)
Not remember pre-pregnancy weight (kg)	228	48.7	
Pre-pregnancy BMI (kg/m^2^)	240	51.3	25.6(± 5.7)
Pre-pregnancy BMI (kg/m^2^) classification			
Underweight (< 18.5)	15	6.6	
Normal (18.5–24.9)	120	47.1	25.5 (SD ± 6.3)
Overweight (25–29.9)	57	25.1	
Obese (≥ 30)	48	21.1	
Measured Height (cm)	468	100.0	159 (SD ± 6.3)
Weight during pregnancy (kg)	468	100.0	68.9(SD ± 12.9)
BMI during pregnancy (kg/m^2^)	468	100.0	27(SD ± 12.9)
MUAC			
< 28 cm = Normal	299	63.9	27 (SD ± 3.8)
≥ 28 cm = Above normal	169	36.1	

Most of the women delivered through normal vaginal means (92.6%, *n* = 363) and 7.4% (*n* = 29) through caesarean section at a mean gestational age of 38 weeks (SD ± 1.7) where 98.5% delivery at ≥ 37 weeks of gestation. Miscarriages and abortion were not observed in the study while stillbirth was observed in 0.5% (*n* = 2) of the women, neonatal death (1.3%, *n* = 5), and 8.2% (*n* = 32) had macrosomic babies (≥ 4 kg) at birth (Table 3).

**Table 3:** Selected pregnancy outcomes

Variables assessed	Frequency	Percent	Mean (SD)
Gestational age at delivery (*n* = 392)			
≥ 37 weeks	386	98.5	
< 37 weeks	6	1.5	38(SD ± 1.7)
Birth modality (*n* = 392)			
Caesarean section	29	7.4	
Normal delivery	363	92.6	
Miscarriages/abortions (*n* = 392)			
Yes	0	0.0	
No	392	100.0	
Stillbirth (*n* = 392)			
Yes	2	0.5	
No	390	99.5	
Neonatal death (*n* = 390)			
Yes	5	1.3	
No	385	98.7	
Childs weight at birth (*n* = 385)			
< 2.5 kg (Low weight)	16	4.1	
2.5–3.9 kg (Normal weight)	337	87.7	3.3 (SD ± 0.5)
≥ 4 kg (macrosomic)	32	8.2	

Binary logistic regression was done for each factor’s association with GDM. The cut off point for body fat percentage was determined using the ROC and found to be body fat of ≥ 38%. From the univariate logistic regression model, potential predictors for development of GDM among pregnant women included; high MUAC, body fat percentage, previous stillbirth, delivery mode (Caesarean section), family history of T2DM, previous delivery to macrosomic babies at *P* < 0.05 (Table 4). The analysis involved many parameters which were found to have no association with GDM including self-reported pre-pregnancy weight and weight during pregnancy (measured at the time of testing).

**Table 4:** Univariate analysis of risk factors associated with GDM

Variables	B	COR	CI	*P*-value
Mother’s age ≥ 25yrs	0.005	1.005	0.959–1.052	0.846
Pre-pregnancy BMI	0.023	1.024	0.967–1.084	0.42
BMI during pregnancy	0.087	0.55	0.28–1.02	0.85
Macrosomic (≥ 4 kg)	1.095	2.989	1.631–5.478	< 0.001*
T2DM history	1.661	5.265	2.879–9.627	< 0.001*
MUAC ≥ 28 cm	0.467	1.596	1.441–1.767	< 0.001*
Body fat ≥ 38%	0.498	1.645	1.460–1.855	< 0.001*
Parity ≥ 4 births	0.093	1.098	0.456–2.644	0.835
Preterm delivery	0.132	1.141	0.324–4.015	0.837
Stillbirth	1.329	3.779	1.343–10.628	0.012*
Neonatal death	-19.439	0.000	0.000–0.004	0.999
Hpertension during pregnancy	0.321	1.378	0.731–2.600	0.327
Reported pre-pregnanacy hypertension	-19.355	0.001	0.000–0.005	0.999
Alcohol intake	0.095	1.100	0.184–6.566	0.917
Presence of glucose in urine	1.015	1.111	0.103–12.037	0.931
**Current pregnancy outcomes**				
Multiparity	-19.442	0.000	0.00–0.222	0.999
Delivery mode (Caeserean section)	0.793	2.209	1.088–5.505	0.0089*
Macrosomic	1.058	2.882	1.238–6.707	0.014*

The word “No” was used as reference in categorical variables

*Abbreviations*: *CI* Conidence Interval, *COR* Crude OR odd ratio, *B* Beta which is the regression coeficiency

^*^Significant at *p* < 0.05

**Fig. 1:**
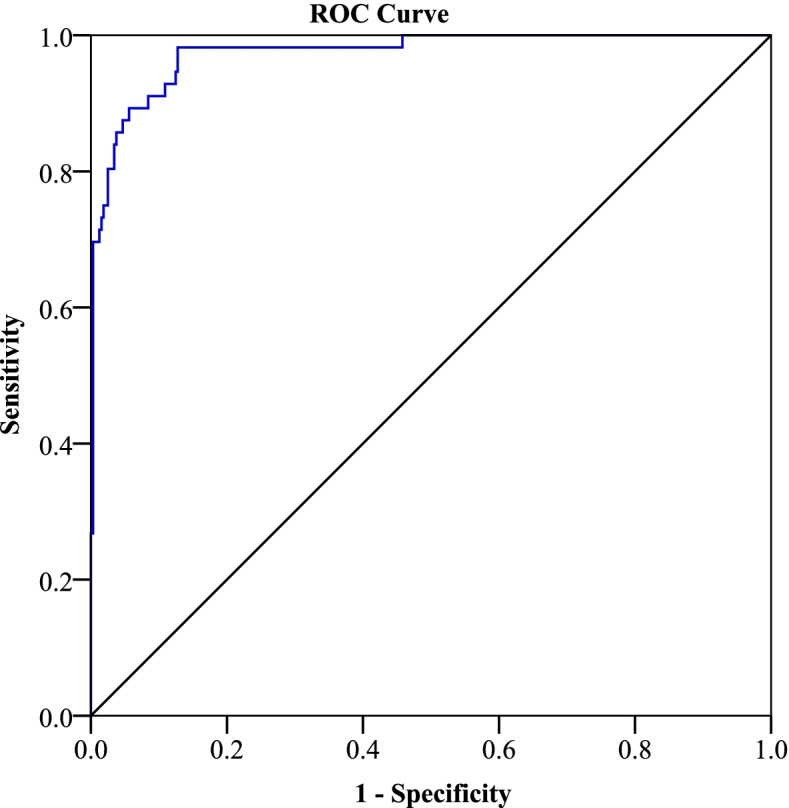
ROC for performance of the risk score model

Multivariate analysis shown that, GDM was significantly associated with MUAC ≥ 28 (AOR 1.281 95% CI 1.080–1.575), body fat percentage (AOR 1.77, 95% CI 1.370–2.294), family history of T2DM (AOR 8.34, 95% CI 1.907–36.43), and previous or current delivery to macrosomic babies (AOR 7.99, 95% CI 1.947–32.786). During the backward elimination; stillbirth, and delivery mode (caesarean section) were removed from the model. The significant variables were used to develop a risk score model to identify women at risk of GDM and the risk for GDM increased among woman with 2 to 49 scores (Table 5). The model performed well in the selected ANC with an AUC of 0.97(95% CI 0.96–0.99, *p* < 0.001) (Fig. 1), sensitivity of 0.98, specificity of 0.46 as well as PPV of 0.68 and NPV of 0.97 at a selected cut off of 0.2. The threshold of 0.2 was selected as cut off for performance of the risk score model to reduce the number of false negatives. Moreover, the regression has the pseudo-R squared of 80% which implies that the model is a good predictor for GDM (Table 5).

**Table 5:** Multivariate analysis and the risk scores

Variables	n	B	A OR	CI	*P*-value	B × 10
Macrosomic delivery in index pregnancy	29	2.000	7.990	1.947–32.786	0.004*	20
History of T2DM	56	2.121	8.336	1.907–36.428	0.005*	21
MUAC ≥ 28 cm	176	0.245	1.281	1.080–1.575	0.019*	2
Body fat ≥ 38%	94	0.572	1.773	1.370–2.294	< 0.001*	6
**Total points**						**49**
R^2^ of 0.803 and ROC 0.971		NA	NA	0.955–0.993	< 0.001*	NA

Significant *p* < 0.1

*Significant at *p*<0.05

The abbreviation *AOR* Adjusted odd ratio, *CI* Confidence interval, *NA* Not applicable

**Fig. 2:**
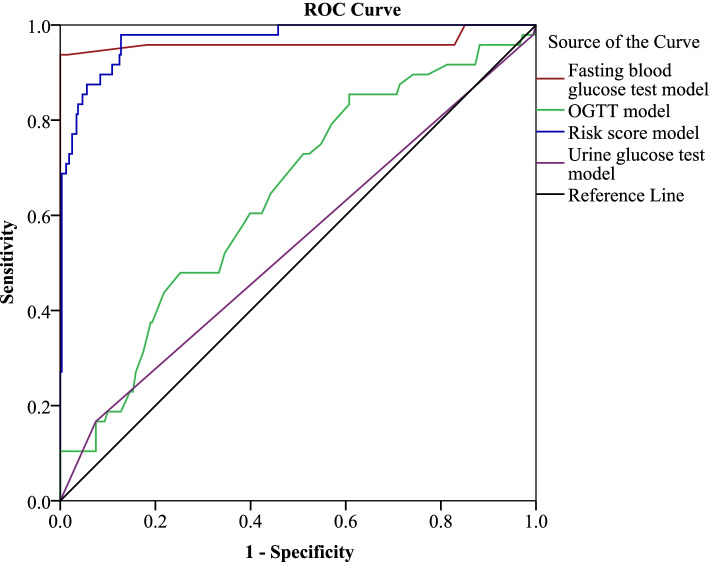
Performance of the models

The performance of the risk score model was significantly higher with AUC of 0.97 (95%CI 0.95–0.99, *p* < 0.001) compared to that of the fasting glucose test with AUC of 0.96 (95%CI 0.92–0.99, *p* < 0.001), and OGTT with AUC of 0.64 (95% CI 0.56–0.72, *p* = 0.002). Urine glucose test model performed poorly with an AUC of 0.54 (95% CI 0.45–0.63, *p* = 0.38) meaning that it could not discriminate women with and those without GDM (Fig. 2).

The developed risk score was simplified into a checklist which involved family history of T2DM, body fat ≥ 38%, MUAC ≥ 28 cm and macrosomic delivery in previous pregnancies. In this case, having one or more of the risk factors indicated in the checklist exposes a woman to be at risk of developing GDM (Table 6).

**Table 6:** Risk factors checklist

	**Risk factors for GDM**
1	Family history of T2DM
2	Delivery macrosomic babies (≥ 4 kg)
3	Body fat ≥ 38%
4	MUAC ≥ 28 cm

## Discussion

The current study was conducted among pregnant women in urban areas of Arusha District to develop a simplified method for identification of women at risk of GDM in Tanzania’s ANC settings. The developed risk score involved maternal and clinical characteristics such as MUAC ≥ 28 cm, body fat ≥ 38%, family history of T2DM and macrosomic delivery (≥ 4 kg) babies at birth in previous pregnancies. Healthcare interventions on screening and managing GDM can target women with these risk factors to reduce the prevalence of GDM and future T2DM.

The developed risk score was found to perform well with an AUC of 97%, meaning that it can strongly discriminate the randomly selected women with GDM from those not experiencing the condition. The model was found to perform well even when body fat percentage was excluded as the body fat analyzer machine is not readily available in most antenatal care clinics. The risk score was compared with fasting, OGTT and urine glucose test models and found to perform better, followed by the fasting and OGTT models while urine glucose test model was not valuable. This implies that, although a urine test for glucose is the most common used method in Tanzanian ANC program [26] it is insensitive and may cause a large proportion of women with or at risk of GDM to remain undiagnosed. Another study done in Ghana reported that the presence of risk factors followed by OGTT and fasting plasma glucose models were more sensitive compared to glycosuria, random blood glucose and glycated hemoglobin, which were highly insensitive, diagnostically insufficient, and misses majority of the cases [36].

The developed risk score has an ability to identify 98% of the women with positive result and 46% of truly negative women meaning that, it can correctly identify most of the women with or at risk of GDM in these ANC settings. This allows additional testing to few women for further actions to decrease unnecessary screening, reduce costs, inconveniences and promote efficient use of the scarce resources to enhance evidence-based treatment practices. Several similar studies have reported that, selective screening strategies perform well in reducing unnecessary testing and increase early identification of women with or at risk of GDM [30–33, 50–52]. These results encourage the use of selective screening in areas with limited resources.

The already published selective screening strategies have been developed using variety of risk factors for example a developed selective screening tool in Tanzania involved; MUAC ≥ 28 cm, stillbirth, and family history of diabetes, with the ability to identify 69% of the GDM women [35]. There is a slight difference of the previous model with the one developed in the current study due to differences in diagnosis criteria used to identify women with GDM. Furthermore, the current study included more risk factors such as pregnancy outcomes in the index/current pregnancy and body fat percentage which could replace pre-pregnancy BMI, although not very well explored.

Although BMI is commonly used in assessing nutritional status in pregnancy, it does not distinguish between fat and lean body mass [38]. Also, most of the women initiate antenatal clinic (ANC) late and start their pregnancy without knowing their body weights making it difficult to estimate their BMI and weight gain during pregnancy which is strongly correlated to fat mass changes [39]. In addition to body fat and lean body mass, the fetal mass and amniotic fluid contain an unknown part of the total body mass of the mother [39]. On the other hand, MUAC is a relatively simple measure, that may be used instead of BMI due to its relative stability during the course of pregnancy and its high correlation with pre-pregnancy BMI [41, 42]. Furthermore, MUAC does not need complex calculations and expensive equipment, such as height charts and scales and can readily be performed on a serious ill patient who cannot even standup [53]. Beyond these two standard measures, bioelectrical impedance analysis (BIA) for determining body fat percentage is increasingly recognized as a safe, accurate and reliable method for assessing nutrition status [40]. Although BIA is not readily available in most ANC in Tanzania, it is an interesting new technology which may be explored further to provide more objective measures with more evidence.

On the other hand, Caliskan et al*.* [30] developed a risk score in Turkish population which included maternal age, pre-pregnancy BMI, and first-degree relatives with diabetes mellitus, a prior macrosomic fetus (> 4000 g), and adverse outcomes in previous pregnancies. Their score showed a good performance where the number of women to be screened decreased by 63% and diagnosing 85% of cases with GDM. This supports our results partly by including first-degree relatives with diabetes mellitus, a prior macrosomic fetus (> 4000 g), and adverse outcome in the previous pregnancies but it is somehow different as it included BMI which was difficult to determine in our setting. Other studies done in Nigeria and China were also contrary to our findings reporting that the pre-gestational BMI > 25 kg/m^2^ was a determinant of GDM [54] This varied from our findings as most of the women could not recall their pre-pregnancy weight and started ANC at an average of 18 weeks of gestation which made it indeterminant during pregnancy. Instead, the current study used body fat percentage in addition to MUAC as a proxy for BMI because it can easily be measured during pregnancy and post-delivery. Also, body fat percentage is a good indicator of fat deposition compared to BMI which may be affected by weight of the fetus and the fluids which accumulate during pregnancy. Similar study conducted in China reported that the percentage body fat was the strongest risk factor for gestational diabetes after adjusting for pre-pregnancy BMI [55]. A study done in India reported that the estimation of weight for determining BMI may be susceptible to certain bias as it is partly based on self-reported weight or weight measured at first antenatal care visit leading to over-or under-estimation of BMI [54].

Although our risk score shown to perform well, several studies have reported that risk factors have poor predictive value and fail to identify a large proportion of women with GDM [34, 36, 56]. Another review was done to validate 12 published GDM risk scores and reported that they performed only moderately, hence calling for more research to be done before putting the scores into practice [57, 58]. In line with this, some meta-analysis suggests that irrespective of the method used, risk factors do not identify women with GDM well [28] but it is still important to consider these selective screening approaches as they can help in early identification of women at risk for timely management especially in resource limited areas.

Universal screening is highly recommended given availability of financial, material, space, and human resources; however, implementing multiple testing during pregnancy for all women is not only costly, but operationally challenging [54]. This makes selective screening using maternal and clinical characteristics to be important. Another study in South Africa reported that, although universal screening and diagnosis of GDM are widely advocated as a strategy to promote appropriate treatment and improve pregnancy outcomes, it is not feasible in many low- and middle-income countries. As a result, many countries use risk factor-based selective screening [59].

For operationalization, the developed risk score was simplified into a risk factors checklist, for the health care system to integrate it into the ANC services from the point of entry with history taking, throughout counselling and regular education programs. This can increase knowledge about GDM as a risk for poor pregnancy outcomes. This tool can be effective if it is used at the first ANC visit, and in subsequent visits as some of the risk factors can arise at the middle or late stages of pregnancy. When a woman is identified having one or more of the risk factors in the checklist, can be referred to the doctor for more actions to be taken because, each explanatory variable was found to have independent significant association with GDM. This helps to give priority to high-risk women when resources are limited while planning for universal screening. The developed tool can also be used by the women for self-identification even before pregnancy to enhance self-care seeking behaviors for proper preconception preparations.

### Limitations of the study

The results from this study are promising however, the recalled pre-pregnancy weight may not be reliable hence, the prevalence of pre-pregnancy overweight and obesity need to be interpreted with care. The study also used body fat percentage in the development of the model which is a new parameter for more exploration however, the bioelectrical impedance analyzer is not readily available in our ANC settings.

## Conclusion and recommendations

The developed risk score was found to perform well hence, it was simplified into a risk factor checklist for easy interpretation and application by women themselves and health care providers. This tool involved family history of T2DM, macrosomic delivery, high body fat deposition and large MUAC. Therefore, it can be used when resources are limited to give priority to high-risk women while planning for universal screening strategy. Furthermore, large longitudinal research is necessary for cost-effective analysis as well as validation of the developed selective screening strategy prior to its implementation in clinical settings.

## Data Availability

The datasets used during the current study will be available on reasonable request to the corresponding author. This is because the data set contains other data that have not yet been analyzed.

## Abbreviations

MUAC: Mid-Upper Arm Circumferences
GDM: Gestational Diabetes Mellitus
BMI: Body Mass Index
CI: Confidence Interval
OR: Odd Ratio
WHO: World Health Organization
ADA: American Diabetes Association
IDF: International Diabetes Federation
